# Non-invasive brain stimulation can induce paradoxical facilitation. Are these neuroenhancements transferable and meaningful to security services?

**DOI:** 10.3389/fnhum.2013.00449

**Published:** 2013-08-14

**Authors:** Jean Levasseur-Moreau, Jerome Brunelin, Shirley Fecteau

**Affiliations:** ^1^Faculté de Médecine, Centre Interdisciplinaire de Recherche en Réadaptation et en Intégration Sociale, Centre de Recherche del'Institut Universitaire en Santé Mentale de Québec, Université LavalQuebec City, QC, Canada; ^2^Centre Hospitalier le Vinatier, Université de Lyon, Université Claude Bernard Lyon IVilleurbanne, Bron, France; ^3^Berenson-Allen Center for Noninvasive Brain Stimulation, Beth Israel Deaconess Medical Center, Harvard Medical SchoolBoston, MA, USA

**Keywords:** non-invasive brain stimulation, motor function, cognitive function, transcranial magnetic stimulation, security, transcranial direct current stimulation (tDCS), neuroenhancement

## Abstract

For ages, we have been looking for ways to enhance our physical and cognitive capacities in order to augment our security. One potential way to enhance our capacities may be to externally stimulate the brain. Methods of non-invasive brain stimulation (NIBS), such as repetitive transcranial magnetic stimulation (rTMS) and transcranial electrical stimulation (tES), have been recently developed to modulate brain activity. Both techniques are relatively safe and can transiently modify motor and cognitive functions outlasting the stimulation period. The purpose of this paper is to review data suggesting that NIBS can enhance motor and cognitive performance in healthy volunteers. We frame these findings in the context of whether they may serve security purposes. Specifically, we review studies reporting that NIBS induces paradoxical facilitation in motor (precision, speed, strength, acceleration endurance, and execution of daily motor task) and cognitive functions (attention, impulsive behavior, risk-taking, working memory, planning, and deceptive capacities). Although transferability and meaningfulness of these NIBS-induced paradoxical facilitations into real-life situations are not clear yet, NIBS may contribute at improving training of motor and cognitive functions relevant for military, civil, and forensic security services. This is an enthusiastic perspective that also calls for fair and open debates on the ethics of using NIBS in healthy individuals to enhance normal functions.

## Introduction

For centuries, we have been trying to improve our motor and cognitive performance in order to augment our security against predators, including our fellow human beings. Numerous ways have been explored to surpass limitations of the human body (e.g., physical training, education, technology, religion). The discovery of the electric neuronal transmission in the early 1800s' has reinforced the belief that one way to enhance motor and cognitive abilities may be to stimulate the brain using electric currents. Considerable progress in modifying electric neuronal activity non-invasively in living humans has been made in the recent years, making it now possible to modulate behaviors. Two of the modern non-invasive brain stimulation (NIBS) methods are the repetitive Transcranial Magnetic Stimulation (rTMS; for a review see Sandrini et al., 2011) and the recently re-discovered transcranial Electrical Stimulation (tES; for a review see Utz et al., 2010; Jacobson et al., 2012). They are now widely used in cognitive neuroscience to study and modulate human behaviors in pathological and normal conditions. Indeed NIBS can be used to characterize causal relationships between brain networks and behaviors. The brain region that is targeted with NIBS is often chosen based on lesion work and imaging data (e.g., functional MRI) associating a given function with a specific brain network. The general hypothesis postulates that NIBS applied over a specific brain region will modulate level of performance of its associated underlying behavior(s). We can impair and improve normal behavioral performance in healthy individuals with NIBS. When we induce a deficit, this phenomenon is called virtual lesion. When such modulation leads to a functional enhancement, this phenomenon is called paradoxical facilitation. Paradoxical facilitation was first described in patients with brain lesions who performed better than normal subjects on certain tasks (for a review see Kapur, 1996). For example, it has been shown that patients with a right hemisphere lesion displayed shorter response time (RT) than healthy subjects at an attentional task (Ladavas et al., 1990). More recently, it has been reported that NIBS can induce paradoxical facilitation in healthy adults. For instance, normal behavioral performance of healthy subjects can be enhanced following a single session of rTMS or tES. The goal of this paper is to review data indicating that NIBS can promote motor and cognitive functions in healthy volunteers. Further, we frame these data in the context of whether they may benefit security purposes. Specifically, we will discuss how modulation of motor and cognitive functions with NIBS may promote existing training in security services (e.g., military, police).

## Overview of NIBS techniques

**Figure 1:**
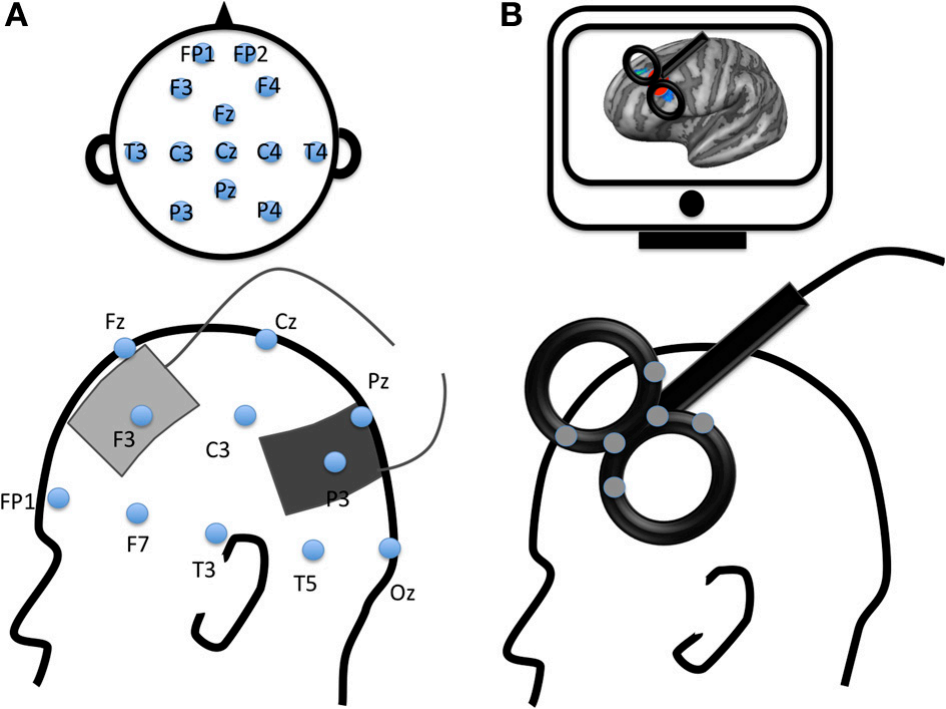
**Examples of electrode montage and coil location for two modern non-invasive brain stimulation techniques. (A)** Example of transcranial Electrical Stimulation electrodes montage with anode (gray) applied over the left dorsolateral prefrontal cortex and cathode (black) applied over the left parietal cortex according to 10–20 EEG international system; **(B)** Example of transcranial magnetic stimulation location with a figure-8 coil applied over the left dorsolateral prefrontal cortex (DLPFC). The gray spots over the transcranial magnetic stimulation coil represent navigator markers, the coil is placed over the DLPFC according to subject's MRI (on a computer screen, top of the figure).

The principle of rTMS is based on Faraday's Law of electromagnetic induction. Brief current pulses are delivered through a coil placed on the subject's scalp (see Figure 1A). This generates a magnetic field that penetrates the scalp and skull, inducing a weak electrical current in the brain. rTMS can induce effects that outlast the stimulation period. Low frequencies rTMS (= 1 Hz) are known to decrease activity, whereas higher frequencies are assumed to increase activity of the targeted brain area. rTMS can also modulate activity of brain regions interconnected with the targeted area (Hoogendam et al., 2010). Specific mechanisms of these changes remain to be fully determined, but they are widely believed to reflect changes in synaptic potential by modulating depolarization or hyperpolarization states of neurons, leading to changes in long-term depression-like and long-term potentiation-like plasticity.

The principle of tES is quite different from that of rTMS. tES consists of applying electrodes on the subject's scalp (see Figure 1B). A weak transcranial Direct Current (tDCS), slow oscillatory Direct Current (so-tDCS, o-tDCS, or tSOS) or Alternating Current (tACS) flows through the brain between the anode and the cathode electrodes. This current flow modulates neural activity in the targeted area(s) as well as connectivity within an interconnected network (Keeser et al., 2011). The effect of tDCS can outlast the stimulation period. The anode is known to increase excitability of the targeted area and the cathode to inhibit it (Nitsche et al., 2007). Although the exact mechanisms underlying tDCS effects remain unknown, pharmacological studies have highlighted changes in resting neuronal membrane potential and synaptic modifications linked to glutamatergic (NMDA-receptor) and GABAergic activity (for a review see Stagg and Nitsche, 2011). These findings were recently supported in a study using Magnetic Resonance Spectroscopy reporting GABA and Glutamate changes following NIBS (Clark et al., 2011).

The brain area can be targeted non-invasively with rTMS or tES on the subject's scalp based on the 10–20 EEG international system, an anatomical, or a functional MRI image (Figure 1).

Specific mechanisms of how NIBS induces paradoxical facilitation in healthy individuals are not completely understood yet. Most researchers agree that from a neurophysiologic perspective, NIBS enhances behavioral performances by modulating a dynamic distributed brain network. From a conceptual perspective, three non-mutually exclusive frameworks have been proposed: the entrainment theory, the stochastic resonance model, and the zero-sum theory (for a review see Pascual-Leone et al., 2012). The entrainment theory posits that the brain can be brought into an oscillatory natural state that is known to be associated with a particular function. According to the entrainment model, NIBS mimics brain oscillations and has an effect by entraining the brain's natural state. For instance, applying slow oscillatory tDCS during sleep induced an increase in slow wave sleep and promoted memory in a frequency-specific manner (Marshall et al., 2004). The stochastic resonance model supposes that small amounts of noise injected into a system promote low-level signals leading to enhanced functions within this system. For instance, TMS at low intensity applied over the visual cortex (V5/MT) facilitated detection of weak motion signals, whereas higher intensities impaired detection of stronger motion signals (Schwarzkopf et al., 2011). Finally, the zero-sum theory posits that the brain has a finite power processing. According to this model, if NIBS induces a paradoxical facilitation, the opposite effect will also be observed that is a detrimental behavioral impact. For example, low frequency rTMS applied over the parietal cortex enhanced target detection in the ispilateral visual hemi-field and worsened detection in the contralateral visual hemi-field (Hilgetag et al., 2001).

## Studies using NIBS to induce paradoxical facilitations

We will here describe studies indicating that NIBS can enhance performance of healthy subjects on motor and cognitive tasks (attention, impulsivity, risk-taking, working memory, planning, and deceptive capacities).

### Effects of NIBS on motor functions

The first application of TMS was on the human motor cortex (Barker et al., 1985); and the use of NIBS to promote motor functions in healthy subjects likely represents the richest literature on facilitations induced by rTMS or tES. We will here present studies reporting that NIBS can induce paradoxical facilitation of motor functions in terms of precision, speed, strength, acceleration endurance, and execution of daily motor task. The majority of these NIBS studies targeted the primary motor cortex (M1), a region known to be involved in motor control (for a review see Schieber, 2001) and motor sequence learning (Penhune and Steele, 2012).

#### Effects of NIBS on motor precision

Studies tested the ability of NIBS to enhance precision of motor functions in healthy subjects. Buetefisch and colleagues tested the effects of low frequency rTMS applied over the left M1 on precision at motor pointing tasks. They used tasks requiring lower and higher demand of precision for both hands (i.e., ipsilateral and contralateral to the stimulated left M1). Participants receiving active rTMS were more accurate in the task demanding higher level of precision for both hands (with greater accuracy for the ipsilateral than the contralateral one), compared to when they received sham stimulation (Buetefisch et al., 2011). For the lower demand level of precision, no difference in precision was observed between active and sham stimulation conditions. Moreover, Matsuo and colleagues tested the precision in a circle-drawing task before and after healthy volunteers received either active or sham tDCS over the right M1. They found that participants receiving anodal tDCS displayed greater precision of the non-dominant-hand movement. No change in precision was observed when subjects received sham stimulation (Matsuo et al., 2011). Also, these enhanced motor abilities (i.e., deviation area and path length of the task) were observed up to 30 min after the end of the stimulation session (Matsuo et al., 2011).

#### Effects of NIBS on motor learning

Nitsche and colleagues investigated the effects of tDCS on implicit motor learning using a modified version of the *Serial Reaction Time Task* (SRTT). In this task, participants are instructed to respond as fast as possible on a response pad with four buttons to the apparition of a dot on a computer screen in one of the four positions (each button have to be pushed with a different finger of the right hand). Anodal tDCS was applied in separate groups of participants to different regions contralateraly to the performing hand: M1, premotor, and prefrontal cortices. Participants receiving anodal tDCS over M1 were faster at executing implicitly learned sequences compared to participants receiving tDCS over the premotor or prefrontal areas (Nitsche et al., 2003). This effect was replicated with rTMS. Healthy subjects who received low frequency rTMS over M1 were faster at executing a learned sequence movement with the hand ipsilateraly to the stimulated M1 without affecting performance with the contralateral hand as compared to rTMS applied to the contralateral M1, ipsilateral premotor area, or vertex (Kobayashi et al., 2004). This effect was reported for both M1, with a greater effect for the right M1. The authors reported no effect on accuracy as measured by error rate. The improvement of ipsilateral motor accuracy following 1 Hz rTMS over M1 can outlast the stimulation period up to 30 min (Avanzino et al., 2008). Similar findings were reported using high frequency rTMS applied over the right M1 in right-handed subjects. Subjects were faster and more accurate to execute a learned complex motor task with their left (non-dominant) hand when they received active rTMS as compared to when they received sham stimulation (Kim et al., 2004). Vines et al. (2008) investigated the effects of tDCS in right-handed healthy participants in a finger sequence performance task. They studied four stimulation conditions: anodal tDCS over the non-dominant M1 coupled with cathodal tDCS over the dominant M1; anodal tDCS over the dominant M1 coupled with cathode over contralateral supraorbital region; anodal tDCS over the non-dominant M1 coupled with cathode over the contralateral supraorbital region, and sham tDCS. The anode applied over the non-dominant M1 coupled with the cathode over the dominant M1 enhanced motor performance in the contralateral (left) hand. Performance was measured by the total number of correct responses calculated as the mean percentage of change in the total number of correct sequential keystrokes at the finger-sequence performance task. The three other stimulation conditions did not lead to significant changes.

#### Effects of NIBS on muscle might

So far we discussed studies indicating that NIBS can improve motor accuracy, learning, and speed. Other studies suggested that NIBS can also promote motor strength, acceleration, and endurance. This has been shown in upper and lower body parts. Tanaka et al. (2009) investigated the impact of tDCS on leg motor strength at a *Pinch Force Test* in healthy subjects. They found that participants receiving anodal tDCS over the right M1 coupled with cathodal tDCS over the left supraorbital area displayed greater strength compared to those receiving cathodal tDCS over the right M1 coupled with anodal tDCS over the left supraorbital area and sham stimulation. Moreover, these effects outlasted the stimulation period by 60 min. Teo et al. (2011) studied the effects of intermittent theta burst stimulation (iTBS; known to increase excitability) over M1 on movement acceleration. They found that iTBS significantly increased peak acceleration of the thumb abduction movement compared to baseline performance. Cogiamanian et al. (2007) explored the effects of anodal tDCS over the right M1 coupled with cathodal tDCS over the right shoulder on muscular endurance in healthy subjects using a paradigm requiring submaximal isometric contraction of the left elbow flexor. They found that, compared to opposite electrode arrangement or sham conditions, anodal tDCS significantly increased endurance of participants (maximum voluntary contraction).

#### Effects of NIBS on execution of daily motor task

Boggio and colleagues investigated the effect of tDCS on motor performance at the *Jebsen Taylor Hand Function Test* (JTHF). The JTHF is a widely used task assessing motor activities often performed in daily life (e.g., picking up small objects and placing them in a can, stacking chequers, moving large light or heavy cans). Right-handed volunteers were faster at completing the JTHF with the left (non-dominant) hand when they received anodal tDCS over the right (non-dominant) M1. There was however no change between active and sham tDCS when performed with the right (dominant) hand (Boggio et al., 2006). In another study, participants who received active tDCS (anode over the right non-dominant M1 coupled with cathode over the dominant M1) combined with unilateral motor training and contralateral hand restraint were faster at the JTHF than those who received sham tDCS combined with unilateral motor training and contralateral hand restraint (Williams et al., 2010). Also, Hummel et al. (2010) tested the effects of tDCS over the left M1 on motor performance measured by the JTHF in healthy subjects. They observed increased overall performance in the time to execute the task in participants receiving active tDCS as compared to when they received sham tDCS. Of note, this study included only elderly participants (mean age of 69 years). Also, these effects were sustained up to 30 min after the end of a single stimulation session. Thus, NIBS appears to decrease speed of motor movement execution.

In sum, NIBS applied over M1 can induce facilitations on various motor aspects such as precision, learning, strength, acceleration, endurance, and execution of daily motor task; and some of these enhancements included hand movements of daily life activities. It also has been proposed that NIBS can be used to enhance motor functions in the context of sportive performance (for a review see Banissy and Muggleton, 2013).

### Effects of NIBS on attentional skills

Attention is a central cognitive process that is considered as a precursor of a large majority of other cognitive functions. Attention can be described as the capacity of sustainably focus cognitive resources on information while filtering or ignoring non-salient endogenous or extraneous information. Attention processes range from the ability to respond to specific visual, auditory, or tactile stimuli to higher cognitive processes of mental flexibility allowing simultaneous responses to multiple tasks. At the brain level, the attention network is a complex set of interactions implying numerous brain regions, especially the frontal and parietal cortices (Petersen and Posner, 2012) and numerous studies have investigated the effects of NIBS on these regions (Fecteau et al., 2006). We will here present studies reporting NIBS-induced paradoxical facilitation of various attentional processes: sustained attention, focused attention, selective attention, attentional switch, and inhibition.

#### Effects of NIBS on sustained attention

Sustained attention is the ability to maintain attention (vigilance) for sporadic critical events during long periods of time (Warm et al., 2008). It elicits a large cerebral network including right and left frontal regions. Nelson et al. (2013) measured the effects of tDCS on vigilance performance in military personnel with an air traffic controller simulator. As compared to sham, active tDCS (anodal over the left DLPFC coupled with cathodal over the right DLPFC, as well as the opposite electrode montage) resulted in enhanced accuracy that is an increased number of correct identified targets and a decreased number of false alarms. However, tDCS also resulted in slower RT. Thus, tDCS can improve sustained attention in setting mimicking work environments such as radar operators.

#### Effects of NIBS on focused attention

Focused attention represents the ability to concentrate the attentional locus toward a specific stimulus. The posterior part of the parietal cortex (PPC) is one of the areas often involved in focused attention, such as detecting a visual target presented in a specific location (for a review see Corbetta and Shulman, 2002). In order to improve focused attention, numerous studies have applied NIBS over the PPC. For instance, a single session of low frequency rTMS applied over the right or left PPC improved detection of stimuli presented ipsilaterally to the stimulated site. The same rTMS protocol also impaired detection of stimuli presented in the contrateral visual field (Hilgetag et al., 2001). These findings were replicated using a single session of low frequency rTMS applied over the right dorsal PPC (Thut et al., 2005). The authors reported enhanced target detection in the right visual field (i.e., shorter RT) and impaired target detection in the left visual field (i.e., decreased accuracy) after rightward cueing in a visual attention detection task. NIBS appears to promote focused attention using stimuli other than visual as well. Anodal tDCS applied over the right PPC coupled with cathodal tDCS applied over the contralateral deltoid muscle improved attention to auditory stimuli presented contralaterally to the stimulation site, the left auditory field (Bolognini et al., 2011). Thus, NIBS can enhance attention in detecting some auditory and visual targets in healthy subjects.

#### Effects of NIBS on selective attention

Selective attention is the ability to focus attentional resources oriented toward a given stimulus despite the presence of distracting or competing stimuli. Amongst the regions presumably involved in selective attention (Petersen and Posner, 2012), the right inferior frontal cortex (IFC) and the PPC have been targeted with NIBS to study selective attention. Selective attention can be studied using the *DARWARS Ambush! Threat Detection Task*. This task was initially designed to train US soldiers bound for Iraq. Subjects are presented with threatening and unthreatening targets that are concealed in realistic virtual situations. They are required to detect threatening targets, such as a bomb under a pile of rocks. Clark et al. (2012) evaluated the effects of tDCS on performance at the *DARWARS Ambush! Threat Detection Task*. Subjects who received active tDCS (anodal over either the right IFC or the right PPC coupled with cathodal over the contralateral upper arm) were significantly better than subjects who received sham tDCS. More specifically, they identified a greater number of correct threatening targets and reported a smaller number of false alarms (i.e., identifying unthreatening targets as threatening ones) at the detection task during and after the stimulation session. They were also increasingly faster to complete the task throughout the four training blocks. The same research team conducted another experiment using the *DARWARS Ambush!* (Falcone et al., 2012). First, they replicated their previous findings: anodal tDCS over the right IFC lead to better identification of threatening concealed objects, lesser number of false alarms, and faster learning curve, as compared to sham tDCS. In addition, they observed that this enhanced performance sustained 24 h after the end of the stimulation period. They conducted a third study with a similar design (Coffman et al., 2012). Here, they replicated their initial findings: subjects who received anodal tDCS over the right IFC were better than those who received sham stimulation (i.e., greater identification of threatening concealed objects, lesser number of false alarms, and faster learning curve), as compared to sham tDCS. The observed enhancements of threat detection with tDCS were associated with increased attention (i.e., alerting attention, decreased RT to detect a cue). Overall, these studies indicate that selective attention can be enhanced by NIBS as shown by improved detection of threats.

#### Effects of NIBS on attentional switch

Attentional switch is the ability to change attentional resources from a given stimulus to another stimulus. It elicits activity in a large cerebral network including some frontal regions (e.g., the medial frontal cortex and the dorsolateral prefrontal cortex; DLPFC) and the pre-supplementary motor area (pre-SMA; Rushworth et al., 2002). Vanderhasselt and colleagues tested the effects of high frequency rTMS over the right DLPFC on attentional switch using a *Task-Switching Paradigm* (Vanderhasselt et al., 2006). In this task, participants had to respond with their hand to a visual stimulus presented on 8 different locations (pressing one of the 8 buttons) and with their foot to an auditory stimulus (pushing a pedal). Participants had to focus their attention to visual stimuli and then to switch their attention when the auditory stimuli occurred. They were faster at switching their attention when they received active rTMS than when they received sham rTMS.

#### Effects of NIBS on inhibition

Inhibition is defined here as the ability to refrain from initiating a response to a stimulus. The right IFC, DLPFCs, pre-SMA, M1, and PPC have been targeted with NIBS to diminish RT and improve accuracy of inhibitory control in healthy subjects. For instance, a single session of high frequency rTMS applied over the left DLPFC significantly decreased RT on incongruent trials at the *Stroop word and color task* as compared to sham stimulation (Vanderhasselt et al., 2007). The Stroop task requires participants to name the font color of the visually presented words. Subjects are usually faster at the congruent than the incongruent condition. The congruent condition consists of presenting the word blue written in blue. The incongruent condition consists for example of presenting the word *blue* written in red. Anodal tDCS over the left DLPFC coupled with cathodal over the contralateral supraorbital area diminished RT at the incongruent condition, compared to sham stimulation (Jeon and Han, 2012).

Inhibition can also be assessed with the *Stop Signal Task* (*SST*). In this task, an external stimulus signals participants to interrupt an already-initiated motor response. The *SST* involves a distributed cerebral network including the IFC, the pre-SMA, and the DLPFC of both hemispheres (Sharp et al., 2010). Studies reported that applying anodal tDCS over the right IFC (Jacobson et al., 2011; Ditye et al., 2012) or the right M1 (Kwon et al., 2013) reduced RT at the *SST* paradigm as compared to sham stimulation. Accuracy at the *SST* can also be improved with NIBS. The number of correct inhibited responses at the *SST* was greater in healthy subjects who received anodal tDCS over the pre-SMA as compared to subjects who received active stimulation over the left M1 (Hsu et al., 2011). NIBS can also enhance these inhibitory skills in healthy subjects in a similar task, the *Conners' Continuous Performance task* (Hwang et al., 2010). Here, participants must press a button each time any letter is presented except the “x” letter. The number of commission errors was reduced when subjects received high frequency rTMS over the left DLPFC as compared to when they received sham stimulation.

The *Flanker Task* is a cognitive paradigm measuring inhibition. Specifically, it characterizes the ability to detect targets in the presence of distracting information. Subjects thus have to inhibit their attention toward distracting stimuli in order to focus their attention on relevant stimuli. Participants who received cathodal tDCS over the right PPC coupled with anodal tDCS over the contralateral supraorbital area were better at detecting targets at this task as compared to subjects who received anodal stimulation over the right PPC coupled with cathodal tDCS over the contralateral supraorbital area and subjects who received sham stimulation (Weiss and Lavidor, 2012). Of note, this NIBS-induced enhancement was not only found in low attentional load, but also in conditions requiring a high level of cognitive process (high-load scenes) when a stimulus is presented along with a great number of distractors.

In sum, NIBS can enhance attentional skills, such as decreasing RT and increasing accuracy at processing visual and/or auditory stimuli in healthy individuals. More specifically, NIBS can improve sustained attention, focused attention, selective attention, attentional switch, and inhibition.

### Effects of NIBS on impulsive behavior

Some studies suggest that NIBS can modulate impulsive behavior. A rich literature in neuroimaging indicate that the DLPFC is critically involved in impulsive behavior (Rorie and Newsome, 2005). Based on this, the DLPFC has been the main targeted region with NIBS. The effects of NIBS on impulsive behavior have been tested using the *Delay Discounting Task*. This task assesses subject's tendencies to prefer smaller, more immediate rewards or larger, delayed rewards. Healthy subjects who received continuous Theta Burst Stimulation (cTBS; known to decrease excitability) over the right DLPFC choose more often larger, delayed rewards than smaller, immediate rewards, as compared to when they received sham stimulation or iTBS over the right DLPFC (Cho et al., 2012). Finally, in an ecological effort, Beeli and colleagues investigated the effect of anodal and cathodal tDCS over the left and right DLPFC on driving behavior (Beeli et al., 2008). They recorded several behaviors in a driving simulator such as distance from driver ahead and speed. They found that participants receiving anodal tDCS, applied either over the left or the right hemisphere, displayed more careful (less impulsive) driving behavior compared to baseline. As seen in attentional inhibition studies, these results suggest that NIBS can also lead to reduced impulsive behaviors.

### Effects of NIBS on risk-taking

Risk-taking is known to elicit activity in several regions, critically including the DLPFC according to neuroimaging studies (Rao et al., 2008). The effects of NIBS applied over the DLPFC in healthy subjects were explored on risk-taking using the *Balloon Analog Risk Task* (BART). In this task, subjects are required to accumulate money by inflating a computerized balloon, whereby they increasingly face the risk of the balloon to explode and loose the accumulated gain. A single session of tDCS with both electrodes over the DLPFCs (i.e., anode placed over either the right or left DLPFC coupled with the cathode over the contralateral DLPFC) led to a more conservative, risk-averse response style (i.e., decreased number of pumps) as compared to sham stimulation and to unilateral active stimulation (i.e., anodal placed over either the right or left DLPFC coupled with cathodal over the contralateral supraorbital area; Fecteau et al., 2007b). NIBS can also induce the opposite behavioral effect at the BART that is increasing risk-taking in healthy subjects. Participants receiving anodal tACS (6.5 Hz) over the left DLPFC coupled with cathodal over the right temporal cortex displayed greater risk-taking (i.e., increased number of pumps from balloons that did not explode) compared to participants receiving sham stimulation and participants receiving anodal right DLPFC tACS coupled with cathodal over the left temporal cortex (Sela et al., 2012).

The effects of NIBS on risk-taking were also investigated with the *Risk Task*. In the *Risk Task* participants have to choose between two options representing different levels of risk and balances of reward. Subjects receiving low frequency rTMS applied over the right DLPFC displayed riskier decision-making style compared to those receiving rTMS over the left DLPFC or sham rTMS (Knoch et al., 2006). NIBS can also decrease risk-taking using the same task. Subjects receiving tDCS (anodal over the right DLPFC coupled with cathodal over the left DLPFC) displayed suppressed risk-taking and decreased sensitivity to reward, as compared to subjects receiving sham tDCS (Fecteau et al., 2007a). Participants receiving active stimulation were also faster at making their choices compared to participants receiving sham stimulation. These studies converge to the suggestion that NIBS can modulate impulsive behaviors and risk-taking.

### Effects of NIBS on working memory

Working memory is a widely investigated cognitive function. Working memory allows to transiently maintain information. It encompasses a large brain network, especially the fronto-temporal network including the DLPFC. Working memory capacities can be assessed by *the Sternberg Task*. This task requires participants to recognize a previously presented item (verbal or non-verbal material) amongst distractors. It has been reported that healthy subjects were faster at *the Sternberg Task* when they received anodal tDCS over the left DLPFC coupled with cathodal tDCS over the right DLPFC as compared to when they received sham tDCS (Gladwin et al., 2012). This effect on *the Sternberg Task* was replicated in a study using high frequency rTMS applied over the left and right DLPFC. Participants were faster (but not more accurate) to perform the task after active (left and right DLPFC) rTMS compared to sham rTMS (Preston et al., 2010). It has also been reported that tDCS over the left DLPFC enhanced working memory as measured by *the backward digit span* (Jeon and Han, 2012). In this task, random sequences of numbers (range 0–9) are verbally presented to participants. The subjects have then to repeat the sequence of numbers in the reverse order. In an ecological effort, working memory can also be studied using an adapted version of the *Object-location learning* paradigm. In this task, subjects had to learn the accurate positions of buildings on a street map by looking at a series of correct and incorrect pairings of buildings (objects) and street map positions (locations). It has been reported that accuracy (i.e., percentage of correct object-location recalls) was improved when subjects received active tDCS (anodal over the right temporoparietal junction coupled with cathodal over the contralateral supraorbital area) as compared to sham tDCS at this task in healthy elderly subjects (mean age of 62 years old; Floel et al., 2012). Interestingly, these effects were found after 1 week (i.e., delayed free recall). It thus appears that NIBS can enhance short term working memory performance in healthy subjects.

### Effects of NIBS on planning

Planning represents the ability to divide behaviors step by step, in a particular order, to reach a specific goal (Unterrainer and Owen, 2006). It involves a large cerebral network including the DLPFC (Unterrainer and Owen, 2006). One well-known paradigm to measure planning is the *Tower of London Task*. In this task subjects are presented with three rods and a number of disks of different sizes which can slide onto any rod. They are invited to preplan mentally a sequence of moves from an initial state to match a goal state (initial thinking phase) and then to execute the moves one by one (execution phase). Some studies indicate that NIBS can improve the overall planning skills at the *Tower of London Task*. Dockery et al. (2009) investigated the impact of tDCS applied over the left DLPFC on this task in a crossover design. Participants were faster (when they received cathodal tDCS) and more accurate (when they received anodal tDCS) to complete the puzzle (preplan and execute) as compared to sham tDCS. Accuracy was calculated as the number of correct solutions divided by the total number of trials. A more recent study reported that cTBS applied over the left DLPFC can diminish the preplan time (initial thinking period) without changing performance at the *Tower of London Task*, compared to when participants received sham stimulation. Of note, iTBS applied over the same brain area lengthened speed of execution at this task (Kaller et al., 2013). Thus, NIBS applied over the DLPFC seems to enhance planning in healthy subjects.

NIBS can also reduce reaction time to solve a problem in an *Analogic Reasoning Task*. This task requires participants to identify analogies between two sets of pictures of colored geometric shapes presented at the same time. Participants were faster at detecting analogies without affecting error rates when they received rTMS over the left DLPFC as compared to when they received rTMS over the right DLPFC and sham stimulation (Boroojerdi et al., 2001).

### Effects of NIBS on deceptive capacities

Deceptive capacities are commonly defined as the abilities to intentionally mislead another individual by falsifying truthful information in a credible way (Vrij et al., 2001). One of the most robust measures to identify deceitful from truthful answers is that deceitful answers are associated with longer onset (Walczyk et al., 2003). Another measure of deceit is the level of guilt as assessed with questions regarding the emotional state (e.g., “Did you feel guilty when lying?”; Caso et al., 2005). Lying elicited activity in several regions, including the DLPFC (Nunez et al., 2005) and the anterior prefrontal cortex (aPFC; Abe et al., 2007). First, it seems that production of lies can be improved (as well as impaired) by NIBS (Karton and Bachmann, 2011). This ability was assessed in a task where subjects have to overtly name the color of a disc (blue or red) presented on a computer screen or lie. The authors investigated the effect of 1 Hz rTMS applied over the right and left DLPFC as compared to the same pattern of stimulation applied over the ispilateral parietal cortex. The authors reported that participants produced less truthful answers after they received rTMS over the left DLPFC compared to when they received stimulation over the parietal cortex. Karim et al. (2010) evaluated the effects of tDCS on deceptive abilities using the *Guilty Knowledge Test*. In this task, subjects participate in a thief role-play in which they are supposed to steal money and then to attend to an interrogation. During the interrogation they have to respond to multi-choice questions, usually consisting of six possible answers; one of which that would only be known by a guilty person, the other five answers being equally plausible to an innocent person. Subjects who received active tDCS (anodal over the left parietal cortex coupled with cathodal over the right aPFC) were better at deceiving than when they received sham tDCS. More specifically, they were faster at lying and they reported lesser guilt. The opposite electrode montage (i.e., anodal over the left aPFC coupled with cathodal over the right parietal cortex) did not modulate deceptive behaviors (Karim et al., 2010). The effects of tDCS on other deceptive abilities were also investigated (Fecteau et al., 2013). Three kinds of stimulation parameters were compared: the anode over the right DLPFC coupled with the cathode over the left DLPFC, the opposite electrode arrangement (anodal over the left DLPFC coupled with cathodal over the right DLPFC) and sham tDCS. Main findings include that compared to subjects who received sham stimulation, those who received active tDCS (anodal over the right or left DLPFC coupled with cathodal over the contralateral region) were faster at recalling memorized untruthful answers. No change in RT was found in these subjects for providing truthful responses. In sum, although data are still limited, they suggest that NIBS may improve some deceptive abilities.

## Discussion

**Figure 2:**
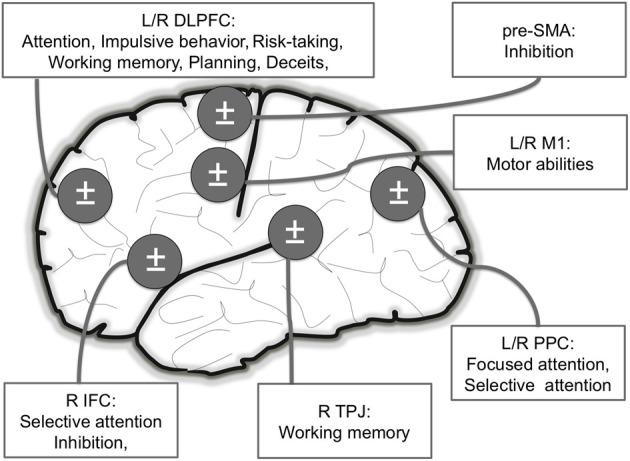
**Main brain areas targeted in NIBS studies inducing motor and cognitive (attention, risk-taking, planning and deceptive abilities) enhancements in healthy volunteers**. L: Left; R: Right; DLPFC: Dorso Lateral Prefrontal Cortex (attention, risk-taking/impulsivity, planning and deceptive abilities); IFC: Inferior Frontal Cortex (attention and deceptive abilities); PPC: Posterior Parietal Cortex (attention); M1: Primary Motor Cortex (motor); TPJ: temporoparietal junction (working memory).

We reviewed here studies indicating that NIBS can improve normal performance in healthy subjects (see Figure 2). Specifically, these improvements were observed for motor abilities (e.g., greater muscular endurance), attentional processes (e.g., faster threat detection), impulsive behavior (e.g., choosing more often larger, delayed rewards than smaller, immediate rewards), risk-taking (e.g., displaying more careful behaviors, diminished or increased risk-taking), memory (e.g., increased working memory load), planning (e.g., enhanced fluid reasoning), and deceptive capacities (e.g., decreased RT in providing deceitful answers).

Interestingly, some of these motor and cognitive processes that can be enhanced using NIBS are already targeted in specific training programs for security purposes. Indeed, some approaches already exist to develop soldiers' motor abilities to emphasize combat readiness. Amongst them, the *Army Physical Fitness Test* is a common program to train physical performance in military. This program trains multimodal aspects of motor performance such as endurance, mobility, strength, and flexibility (Heinrich et al., 2012).

There are also several training programs to enhance cognitive functions for security purposes. Training attention to detect threatening stimuli constitutes one of the highest priorities for security services (see report from the Committee on Opportunities in Neuroscience for Future Army Applications and Council, 2009). Airport security screening staff are trained with *computer-based training* programs to improve their attentional skills in order to enhance their abilities to detect threatening objects in X-ray images (Schwaninger, 2004). As previously discussed, the *DAWARS Ambush!* program was developed to train soldiers to accurately detect threatening objects in realistic environment. Similarly, soldiers are trained to enhance their attentional skills in shooting using the *pop-up target friend or foe* programs (Kelley et al., 2011). In this training program, soldiers have to shoot or refrain from shooting targets representing either friends or foes. Accuracy and RT are trained during specific shooting training. Another training consists of developing automatic behavior to reduce aversive effect of stress on performance for which cognitive control is needed (Leach, 2004). In this way, soldiers are trained to create and follow cognitive automations so-called drills (e.g., *if you are under fire, you find cover*; Delahaij et al., 2006). There is also *The Reid training program* (Jayne and Buckley, 1999), which provides interrogation and interviewing techniques seminars. The goal of this training program is to develop adaptative attentional skills, planning abilities, memory abilities, and appropriate risk-taking. In sum, several of these motor and cognitive skills, as mentioned earlier, can be enhanced with NIBS in healthy subjects. Thus, one might speculate that NIBS may be a promising neuroenhancement tool for security purposes. However, transferability and meaningfulness of these NIBS-induced paradoxical facilitations into real life situations are not clear yet.

### Are NIBS-induced paradoxical facilitations transferable into real-life situations?

Before proposing NIBS as a neuroenhancement tool for security purposes, we have to discuss whether these enhancements may be transferable into real-life situations. Indeed, most of the NIBS-induced facilitation data reviewed here have been collected in laboratory settings. This particular environment using rigorous scientific methods is needed to identify as much as possible the exact changes that are induced by NIBS, not only the improvements, but also potential impairments with controls conditions for instance. This represents an important step toward the development of a new neuroenhancement technique. However, if we want to use NIBS to improve functions relevant in real-life situations, we need to explore whether they can be transferred into real-life.

One avenue to further transferability is to promote the ecological validity of the experimental tasks. Several factors can be promoted to boost the ecological validity of experimental testing. A first factor is how the function is measured. Most functions are measured with computer programs. For instance, target detection can be assessed in laboratory settings using the *Flanker task* (Lavie and Cox, 1997). More recently, target detection has been tested in a more ecological task: the *DAWARS Ambush!* As mentioned earlier, this computer-based program simulates foreign countries environments to train threat detection in war situations (e.g., detect land mines or the safe hidden path used by the enemy to avoid these mines into realistic environment). The effects of NIBS on target detection using the *Flanker* and the *DAWARS Ambush!* paradigms have also been tested. Target detection was improved with active as compared to sham stimulation in healthy subjects at the *Flanker task* (Weiss and Lavidor, 2012) and the *DARWARS Ambush!* (Clark et al., 2012; Falcone et al., 2012). Another example is impulsivity. A common way to test impulsivity level in laboratory settings is with a computer-based task, the SST (O'Brien and Gormley, 2013). Efforts have been made to test impulsivity in more ecological paradigms, such as using a driving simulator (Pearson et al., 2013). The effects of NIBS have been tested on impulsivity on these tasks. Active stimulation as compared to sham stimulation can lead to lower impulsivity level at the SST (Hsu et al., 2011) and at the driving simulator (Beeli et al., 2008). Another example is working memory. A widely used task to characterize working memory and learning is the *Sternberg Task*. In order to assess spatial working memory in a more ecological context, performance of subjects can be assessed using map-learning procedure based on existing maps (Bosco et al., 2004). In such *Street Map Task*, objects are placed on a map and participants have to remember the positions of the objects. The effects of NIBS have been tested on both the *Sternberg Task* and a *Street Map Task*. Results revealed that NIBS improved working memory at the *Sternberg Task* (Gladwin et al., 2012) and at the *Street Map Task* (Floel et al., 2012). These examples are good models to follow to promote the ecological value of laboratory setting without compromising scientific methodological rigor.

In order to promote the effects of NIBS in this population, we need to test the effects of NIBS on ecological tasks and mimic as much as possible external factors that might have an impact, such as performing under stressful situations. Technological advances such as the development of immersive 3D scenarios will certainly optimize smooth translation from laboratory programs into real-life situations. A last point to discuss concerns the generalization of these NIBS-induced improvement at specific task to the whole functioning (global intelligence) as it can be the case with cognitive training (Jaeggi et al., 2010). Now, let's say that in the best-case scenario, NIBS can be transferred into real-life situations. The next question is: *Are these NIBS-induced paradoxical facilitations meaningful for real-life situations?*

### Are NIBS-induced paradoxical facilitations meaningful for real-life situations?

Throughout this paper we presented studies showing paradoxical facilitation induced by NIBS on various motor and cognitive functions. If these NIBS-induced motor and cognitive enhancements are transferable in real-life situations, another question that remains is whether they are meaningful for security purposes. Meaningfulness is defined here as the magnitude and the duration of the effects, in other words *Are they big enough to have a real impact?*

Magnitude of these NIBS-induced facilitations is widely variable. Although statistically significant, whether the magnitude of these enhancements is meaningful for daily-life situations is not clear yet. For instance, Pascual-Leone et al. (2012) estimated a mean reduction of 32 milliseconds from studies using NIBS to improve motor RT. In the specific context of speed shooting performances, ~13 milliseconds would be the difference between elite and rookie police officers (Vickers and Lewinski, 2012). Therefore, an improvement of 32 ms may make a vital difference in the context of a one-on-one gunfight or during aircraft combat (dogfight). This suggested that the magnitude of NIBS-induced enhancements might have a real interest for soldiers and police officers. On the other hand, the magnitude of the enhancement typically observed using NIBS are rather the same as those observed using pharmacological enhancers such as caffeine (Husain and Mehta, 2011). Duration of these NIBS-induced paradoxical facilitations is widely variable across studies, from several minutes to several months (Dockery et al., 2009; Reis et al., 2009). Duration of these effects obviously plays an important role in determining whether these enhancements are meaningful for real-life situations or determining the best timing to stimulate or re-stimulate. Even when tested in laboratory settings in which testing is rigorously controlled, the real duration of these enhancements remains uncertain.

Several factors can influence the magnitude and duration of these paradoxical facilitations, thus ultimately transferability of laboratory findings into real-life situations. These factors can be related to (1) the NIBS device, (2) the brain state, and (3) the behaviors.

1. Factors related to the NIBS device that can influence facilitation include the stimulation parameters. These parameters such as frequency, intensity, number of pulses, and number of sessions can influence the magnitude and duration of paradoxical facilitations. For instance, Iyer et al. (2005) found greater effects with 2 mA than 1 mA on verbal fluency in healthy subjects.
2. Brain state can also influence the effects of NIBS on paradoxical facilitation. State dependency can be defined as the baseline state of brain dealing with many factors such as fatigue, sleep, experience, and personality traits (e.g., Silvanto and Pascual-Leone, 2008; Silvanto et al., 2008). The effects of NIBS can be state dependent. One example comes from tDCS. Slow oscillatory-tDCS (i.e., 0.75 Hz) applied bilaterally over the DLPFC during slow wave sleep increased retention of declarative memory capacities (word pairs previously learned), as compared to sham stimulation. This improvement of memory capacities was associated with an increased sleep depth and slow oscillatory activity (<3 Hz), whereas the power in the faster frequency EEG bands (theta, alpha, and beta) was reduced. In contrast, with the same protocol of slow oscillatory-tDCS, but applied during the wake retention interval, there were no effects on declarative memory (Marshall et al., 2004). Thus, administrating NIBS during a specific sleep phase facilitated sleep-dependent consolidation of declarative memories. This kind of result highlights the importance of state-dependency in NIBS-induced paradoxical facilitation. In other words, in order to optimize NIBS efficacy, we have to determine the best state the subject needs to be before, during and after stimulation. For instance, Kwon et al. (2013) reported that NIBS improved inhibition when it was applied while subjects performed the SST, but it had no effect when it was applied before the SST.
3. Behavioral level at baseline can also influence the effects of NIBS on paradoxical facilitation. Even when performance of a group of subjects is considered normal, within the normal range, some subjects displayed better performance than others (e.g., normal distribution). This baseline level of performance may influence the effects of NIBS. For example, NIBS improved visual working memory skills in low performing subjects, but not in higher performing ones (Tseng et al., 2012).

Age and gender can influence behavioral performance as well as the effects of NIBS. Indeed, baseline performance can vary according to subject's age and gender. Throughout life, our skills naturally change. For example older individuals present slower RT to motion onset than younger ones (Porciatti et al., 1999). Attentional capacities also change with aging (McDowd and Craik, 1988). The same observation has been reported on planning abilities with older adults displaying worse performance at the *Tower of London task* than younger adults (Phillips et al., 2006). Normal aging also affects working memory. For example, it has been reported that older participants displayed both reduced accuracy and slower RT at working memory tasks compared to younger participants (Gazzaley et al., 2005). In sum, it is well-accepted that motor and cognitive performance change through aging (see review from Glisky, 2007). The influence of age on NIBS-induced paradoxical facilitation has not been however extensively investigated yet (for a review see Freitas et al., 2013). One study reported that rTMS induced greater facilitation of inhibition at the *Go/NoGo task* in younger than older adults (age range 28–37 years; Huang et al., 2004), whereas another study reported that NIBS led to greater improvement of motor skills in older than younger participants (age range 56–87 years; Hummel et al., 2010). On one hand, it is possible that NIBS induces larger facilitation in younger than older adults. Indeed, age was reported to correlate negatively with the duration of NIBS-induced neurophysiological effects: longer-lasting effects were found in younger than older healthy subjects. It is speculated that this change in cortical plasticity through aging is linked to normal motor and cognitive decline (Freitas et al., 2013). On the other hand, it is possible that normal performance in older individuals might be easier to improve with NIBS than in younger ones. We could call this motor or cognitive *rejuvenation* that is making older individuals performing as when they were younger.

Gender may also be a considerable factor when using NIBS to induce facilitation in healthy subjects. At the behavioral level, baseline performance can differ according to gender. For example, men are more accurate at a throwing task than women (Moreno-Briseno et al., 2010). Cognitive performance has also been reported different according to gender in numerous functions (for a review, see Zaidi, 2010), such as attentional inhibition (Halari et al., 2005), visual-spatial attention (Rubia et al., 2010), and spatial working memory (Duff and Hampson, 2001). The influence of gender on NIBS-induced effects has not been rigorously studied and remains to be further characterized (Ridding and Ziemann, 2010). Most NIBS studies are not specifically designed to test for gender differences. In sum, further studies are needed to characterize the real influence of several factors, including those related to the device, brain state, behavioral level at baseline, age, and gender on NIBS-induced paradoxical facilitation. Better knowledge of these factors will certainly help to smooth transferability and increase meaningfulness of laboratory setting protocols into real-life contexts.

Another way to improve transferability and meaningfulness of the NIBS induced effects might be to use NIBS as an *add-on* to existing training programs. NIBS may promote capacities that are critical for security purposes. Some studies reported that the combination of motor training and NIBS lead to greater motor improvements than to a single method approach (e.g., physical exercise alone; Bolognini et al., 2009; Williams et al., 2010). This has also been reported in cognition. Combining cognitive training with NIBS resulted in greater effects than single method approach (e.g., stimulation alone). For instance, the combination of a n-back training and active tDCS resulted in greater performance at *the digit span task* than tDCS used as a single method approach and the combination of *the n-back* training and sham tDCS (Andrews et al., 2011). Thus, existing programs developed for security personnel might benefit from combining them with NIBS.

### Ethical concerns of using NIBS-induced paradoxical facilitation in healthy subjects

Although this is out of the scope of this review paper, it is important to mention that this field—inducing paradoxical facilitations with NIBS in healthy subjects—calls for fair and well-balanced discussions on ethics. This discussion should be to some extent in accordance with lines of conduct from the use of other neuroenhancers, such as smart pills (for review Illes and Bird, 2006; Forlini et al., 2013). At this point, whether or not it is ethical to use NIBS as a neuroenhancement tool for security purposes remains an open debate. If it is, another question remains: *Is it safe?*

### Safety concerns of using NIBS-induced paradoxical facilitation in healthy subjects

There are known risks and hypothetical risks associated with the use of NIBS. These risks are reviewed by different groups on the use of NIBS (Wassermann, 1998; Iyer et al., 2005; Rossi et al., 2009). The classic protocol that is considered safe to reduce depressive symptoms in patients with major depression refractory to medications consists of delivering daily session (a session a day, from Monday through Friday) of high frequency rTMS during 3–6 weeks (O'Reardon et al., 2007). Repeated sessions are delivered in order to induce longer lasting clinical benefits. Common side-effects related to this protocol include headaches or cutaneous discomfort.

In healthy subjects, the use of tDCS has been reported to be safe with a single session in 103 subjects (Iyer et al., 2005). However, there are no safety guidelines for the administration of repeated NIBS sessions over a long period of time in healthy individuals. We cannot solely and directly derive them from safety guidelines established for clinical populations. One reason is that the effects of a given NIBS protocol known to be safe (and even salutary) in a clinical population may not be safe in healthy volunteers. For instance, delivering high frequency rTMS over the left DLPFC can alleviate depressive symptoms in patients with depression (i.e., clinical benefit), but can hinder mood in healthy subjects (i.e., would be considered as a side-effect). Hence, we must consider the possibility that a same NIBS protocol might lead to opposite behavioral effects depending on the studied populations.

Regarding the NIBS-induced enhancement studies, another important related aspect that must be taken into consideration is the possibility of incidentally eliciting other effects. For instance, in line with the zero-sum theory principle, rTMS resulted in improved detection of targets in the ipsi- or contra-lateral visual-field and in impaired detection in the opposite visual field (Thut et al., 2005; Buetefisch et al., 2011). NIBS-induced facilitation of motor function can also shift the speed/accuracy trade-off function (Reis et al., 2009; Nelson et al., 2013). This dual effect is not new, nor restricted to the use of NIBS. This speed/accuracy trade-off is commonly observed in cognitive programs (Van Veen et al., 2008). Novice inspectors of aircrafts are trained to detect defects with immersive virtual scenarios. This training leads to increased attentional accuracy, but also to increased RT to detect threatening defects (Sadasivan et al., 2005). We might not be able to prevent or minimize some of these trade-offs yet, but the benefit/risk ratio should be carefully addressed. With regards to hypothetic risks, it is also important to keep in mind some results from the animal literature. It is well-known that animal can develop an addiction to auto-electrical stimulation. This represent an hypothetical risk for humans to develop an addiction to neuroenhancers (Heinz et al., 2012).

## Conclusion

In this article we reviewed experimental data supporting that NIBS can enhance motor (precision, speed, strength, acceleration endurance, and execution of daily motor task) and cognitive functions (attention, impulsivity, risk-taking, working memory, planning, and deceptive capacities) in healthy individuals. Some of these functions are already trained with existing programs for security services. It is thus tempting to speculate that NIBS may serve as a neuroenhancer tool for security purposes. However, numerous questions remain to be answered to do so. We believe that two important questions are (1) *Are these paradoxical facilitations induced in laboratory settings transferable into real-life situations?* and (2) *If they are transferable, are they meaningful for real-life events*? Furthermore, ethical and safety concerns should be carefully addressed.

### Conflict of interest statement

The authors declare that the research was conducted in the absence of any commercial or financial relationships that could be construed as a potential conflict of interest.
